# Thermodynamic Definitions of Temperature and Kappa and Introduction of the Entropy Defect

**DOI:** 10.3390/e23121683

**Published:** 2021-12-15

**Authors:** George Livadiotis, David J. McComas

**Affiliations:** Department of Astrophysical Sciences, Princeton University, Princeton, NJ 08544, USA; dmccomas@princeton.edu

**Keywords:** temperature definition, temperature, kappa distributions, nonextensive entropy

## Abstract

This paper develops explicit and consistent definitions of the independent thermodynamic properties of temperature and the kappa index within the framework of nonextensive statistical mechanics and shows their connection with the formalism of kappa distributions. By defining the “entropy defect” in the composition of a system, we show how the nonextensive entropy of systems with correlations differs from the sum of the entropies of their constituents of these systems. A system is composed extensively when its elementary subsystems are independent, interacting with no correlations; this leads to an extensive system entropy, which is simply the sum of the subsystem entropies. In contrast, a system is composed nonextensively when its elementary subsystems are connected through long-range interactions that produce correlations. This leads to an entropy defect that quantifies the missing entropy, analogous to the mass defect that quantifies the mass (energy) associated with assembling subatomic particles. We develop thermodynamic definitions of kappa and temperature that connect with the corresponding kinetic definitions originated from kappa distributions. Finally, we show that the entropy of a system, composed by a number of subsystems with correlations, is determined using both discrete and continuous descriptions, and find: (i) the resulted entropic form expressed in terms of thermodynamic parameters; (ii) an optimal relationship between kappa and temperature; and (iii) the correlation coefficient to be inversely proportional to the temperature logarithm.

## 1. Introduction

The existence of generalized thermal equilibrium requires the system to be residing in stationary states, i.e., where the (time independent) thermodynamic parameters, such as the temperature and the entropic index, can be well-defined.

Let two arbitrary and initially independent systems A and B interact through a wall permeable to heat transfer, eventually reaching (the generalized) thermal equilibrium. This is a stationary thermodynamic state, where no exchange of energy or entropy is observed [1,2,3], following:

(1) $$\frac{1}{1-\frac{1}{\kappa }S_{A}}\cdot \frac{\partial S_{A}}{\partial U_{A}}=\frac{1}{1-\frac{1}{\kappa }S_{B}}\cdot \frac{\partial S_{B}}{\partial U_{B}},$$

and

(2) $$S_{A+B}=S_{A}+S_{B}-\frac{1}{\kappa }S_{A}S_{B},$$

where we note that (i) the above equations provide, respectively, (a) a generalized thermodynamic definition of temperature (e.g., [1,3,4,5,6,7]), and (b) a partition expression of the entropy (e.g., [8,9]); (ii) the kappa index that parameterizes the formulation of kappa distributions was shown to be equivalent to the notation of the *q*-index that parameterizes the formulations of nonextensive statistical mechanics, under the transformation of their indices *κ* = 1/(*q* − 1) (e.g., see: [5]); and (iii) the Boltzmann constant *k*_B_ is set to 1 throughout the paper.

The temperature is defined through the change of entropy *S*, which is caused by a change of internal energy U; this is formally expressed through the invariant form shown in Equation (1).

(3) $$\frac{1}{T}\equiv \frac{1}{1-\frac{1}{\kappa }S}\cdot \frac{\partial S}{\partial U}.$$

This recovers the classical thermal equilibrium and the Clausius related formulation for *κ→*∞, that is, 1/T≡∂S/∂U [10] (see also: [11,12]. Note: canonical ensemble, the volume, and number of particles of the system is constant, i.e., ∂S/∂U means ${\left(\partial S/\partial U\right)}_{V,N}$).

The entropy partitioning rule of Equation (2) describes a special type of entropy composability; the latter means that the entropy of the total system, which is composed of independent subsystems, can be given as a function of the entropies of the subsystems [13], i.e., $S_{A+B}=f(S_{A},S_{B})$ (see also: [14,15]). This exact form is sometimes called the pseudo-additivity rule because it generalizes the classical entropy additivity rule, that is, $S_{A+B}=S_{A}+S_{B}$, which it recovers for *κ*→∞. Apparently, for systems with correlations this rule does not generally apply, while for special types of correlations it is possible that the additivity may be recovered (e.g., [16,17]).

It has been shown that the pseudo-additivity rule is equivalent to the Tsallis entropy and to the formulation of kappa distributions: First, we recall that Tsallis entropy [18,19,20]) is equivalent to the formulation of kappa distributions (e.g., [5,21,22,23,24]; see also: [25,26,27] and [28,29,30,31,32,33,34,35,36]); indeed, this entropic formulation is maximized under the constraints of canonical ensemble leading to kappa distributions [5], and vice versa, when maximized, the entropic formulation leads to the specific form of kappa distributions [37,38]. Then, it can be easily shown that the Tsallis entropic function obeys to the pseudo-additivity rule [18]. Alternatively, when starting from the pseudo-additivity rule, the entropic function obeying this rule is the Tsallis entropy [3,8,39,40].

The pseudo-additivity rule provides the thermodynamic definition of kappa:

(4) $$\frac{1}{\kappa }\equiv \frac{(S_{A}+S_{B})-S_{A+B}}{S_{A}S_{B}},$$

which provides a measure of the loss (for *κ* > 0) or gain (for *κ* < 0) of entropy after mixing partial systems A and B to compose the combined system.

The expressions in Equations (3) and (4) are stated as the thermodynamic definitions of temperature and kappa, respectively. However, they involve several physical inconsistencies, which we resolve in this paper:

(i) The thermodynamic definition of the temperature is not well-stated since it involves also another independent thermodynamic parameter, the kappa index.
(ii) There is no clear connection between entropic loss and kappa for any individual or composed system, and the same holds for the thermodynamic definition of the kappa.
(iii) There is not an obvious connection with the formalism of kappa distributions and the corresponding kinetic definitions of temperature and kappa index.
(iv) There is no well-determined formulation for the entropy of a combined system with correlations between its subsystems.

The purpose of this paper is to develop explicit thermodynamic definitions of temperature and the kappa index and show their connection with the formalism of nonextensive statistical mechanics and kappa distributions. In Section 2, we interpret the entropy variations that have occurred along the composition of a system; in particular, we define the concept of an “entropy defect” and develop the composition of a system in both discrete and continuous descriptions. We also show the connection of nonextensive entropy characterized by an entropy defect, with the formalism of kappa distributions. In Section 3, we provide consistent thermodynamic definitions of the kappa index and temperature and show their connection with the respective kinetic definitions. In Section 4, we derive the complete formulation of nonextensive entropy in terms of temperature, in both the discrete and continuous descriptions, and show how this form is associated with kappa distributions. In Section 5, we express the nonextensive entropy defect of a system composed by a number of subsystems with correlations in terms of temperature. By maximizing this entropy defect, we also determine a relationship between the independent thermodynamic parameters of kappa and temperature. Finally, Section 6 summarizes and provides the conclusions, while in the Appendix A we provide the basic derived expressions in terms of the entropic *q*-index.

## 2. Entropic Composition of a System

### 2.1. Entropy Defect

While the composition of two, originally independent, subsystems A and B into the combined system A+B is generally completed through an irreversible mixing thermodynamic process, it can be approached by a progressive series of slow quasi-stationary and quasi-reversible (called quasistatic) processes, for which the entropy is additive (e.g., [41,42]). At each procedure, a small portion of entropy *dS* is moved from each partial system to the total, $dS_{A+B}\approx dS_{A}+dS_{B}$, until the completion of all the mixing procedures, obtaining $S_{A+B}\approx S_{A}+S_{B}$.

In this classical approach, there is no consideration of the entropy associated with correlations induced by long-range interactions acting among the particles. When A and B were separated, there was no interaction or correlation between them. Once they are mixed into a combined system characterized by such interactions that can produce correlations (e.g., long-range interactions), then order is added to the system, and thus its entropy decreases. If the long-range interactions and the induced correlations could have somehow disappeared, then an equal amount of entropy would have been added and consumed by the disorder generated by the resolution of these correlations. Measuring the initial *S*_A_, *S*_B_, and final *S*_A+B_ entropies, one may find the missing entropy due to the correlations, *S*_A_ + *S*_B_ – *S*_A+B_. We interpret this missing entropy as an “entropy defect” *S*_def_, that is, the portion of entropy by which the total entropy decreases the sum of entropies of the combined system due to the order generated by the correlations. This amount of entropy can be released at the composition of the system from its constituents. (Note: hereafter, we will refer to the constituents that compose the whole system, which can be either smaller subsystems of particles or individual particles.)

**Figure 1:**
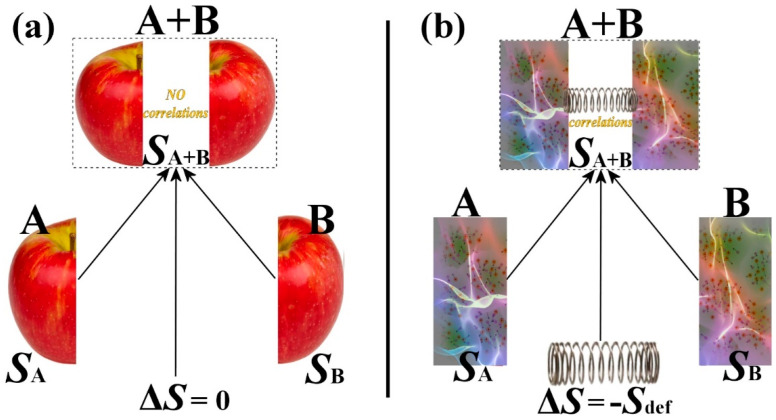
(**a**) The entropy of a system with no correlations among its constituents is additive. (**b**) However, the formation of a system with interactions inducing correlations (such as long-range interactions) requires its entropy to follow a nonadditive rule in terms of the entropies of the subsystems (composability). The difference among the total entropy and the sum of the subsystem entropies equals a missing amount of entropy, the “entropy defect” (Equation (5)), which is a decrease of entropy due to the order generated by the presence of correlations. Later in this paper, we develop the formulae connecting correlations (kinetic definition of the kappa index) with the entropy defect (thermodynamic definition of the kappa index).

As illustrated in Figure 1, the entropy defect is a consequence of the presence of correlations among the constituents of systems. The connection of two subsystems with short-range interactions is not characterized by correlations and thus does not have an entropy defect. On the contrary, the connection of two subsystems with long-range interactions characterized by correlations requires an entropy defect expressed by Equation (5), shown below, which is also consistent with thermodynamics [3].

Thus, we write:

(5) $$S_{A+B}=S_{A}+S_{B}-S_{\mathrm{def}}(S_{A},S_{B}).$$

The simplest construction of the nonadditive and nonlinear term causing the entropic defect would simply be proportional to the initial entropies *S*_A_ and *S*_B_, i.e.,

(6) $$S_{\mathrm{def}}(S_{A},S_{B})=\frac{1}{\kappa }\cdot S_{A}\cdot S_{B}.$$

In fact, this exact formulation, which leads to the entropy partitioning of Equation (2), was shown that can be derived by only assuming the existence of a thermodynamic stationary state, that is, a generalized thermal equilibrium [3]. The constant proportionality term 1/*κ* provides an independent thermodynamic parameter, that is, the kappa index *κ*, (Equation (4)). (In Section 4, we will discuss further the direct connection of correlations with the kappa index, using the findings of [43].)

### 2.2. Composition of a System: Discrete and Continuous Ways

Let the system of entropy *S* be constructed by combining *N* independent elementary subsystems, each of entropy *dS*_ind_. The mixing includes long-range interactions that induce correlations among the subsystems; therefore, there is an entropy defect, *dS*_def_, associated with a kappa index, *κ*. Then, the elementary subsystem interacts and merges with the particle system, changing the entropy of the latter as follows:

(7) $$S_{\mathrm{after}}=S_{\mathrm{bef}}+dS_{\mathrm{ind}}-dS_{\mathrm{def}}(S_{\mathrm{bef}},dS_{\mathrm{ind}}),$$

with

(8) $$dS_{\mathrm{def}}(S_{\mathrm{bef}},dS_{\mathrm{ind}})=\frac{1}{\kappa }dS_{\mathrm{ind}}\cdot S_{\mathrm{bef}}.$$

The collection of a number of *n* independent elementary subsystems can be seen as a classical (extensive) system, without any correlations among the subsystems, whose total entropy is given by:

(9) $$S_{\mathrm{ind}}{}_{n}=n\cdot dS_{\mathrm{ind}}.$$

On the other hand, the collection of the *n* independent elementary subsystems within the (nonextensive) system of entropy *S*, characterized by correlations and thus an entropy defect, is obviously not given by its extensive expression (9). In order to derive the entropy of the system *S* as a function of the elementary element entropy *dS*_ind_, we first need to understand the construction of the system; this can be interpreted either within the continuous or discrete framework. The discrete description follows Equations (7) and (8), which can be written after some calculus:

(10) $$S_{\mathrm{after}}=(1-\frac{1}{\kappa }dS_{\mathrm{ind}})\cdot S_{\mathrm{bef}}+dS_{\mathrm{ind}}.$$

For the continuous description, we substitute in (5), as follows: the elementary subsystem of infinitesimal entropy *dS*_ind_ interacts with the system of entropy *S*; hence, $S_{A}=S$, $S_{B}=dS_{\mathrm{ind}}$ (before) and $S_{A+B}=S+dS$ (after). The entropic change of the system, *dS*, is caused by the elementary subsystem entropies and the entropy defect, i.e.,

(11) $$dS=(1-\frac{1}{\kappa }S)\cdot dS_{\mathrm{ind}},$$

or

(12) $$dS=dS_{\mathrm{ind}}-dS_{\mathrm{def}}\;\mathrm{with}\;dS_{\mathrm{def}}=\frac{1}{\kappa }\cdot S\cdot dS_{\mathrm{ind}}.$$

Equations (10) and (11) express the composition (and decomposition) of a system, by means of its entropy, in a discrete and continuous description, respectively. Both formalisms become trivial in the classical case of no entropy defect, leading into simple additions, i.e., $S_{\mathrm{after}}=S_{\mathrm{bef}}+dS_{\mathrm{ind}}$ and $dS=dS_{\mathrm{ind}}$.

**Figure 2:**
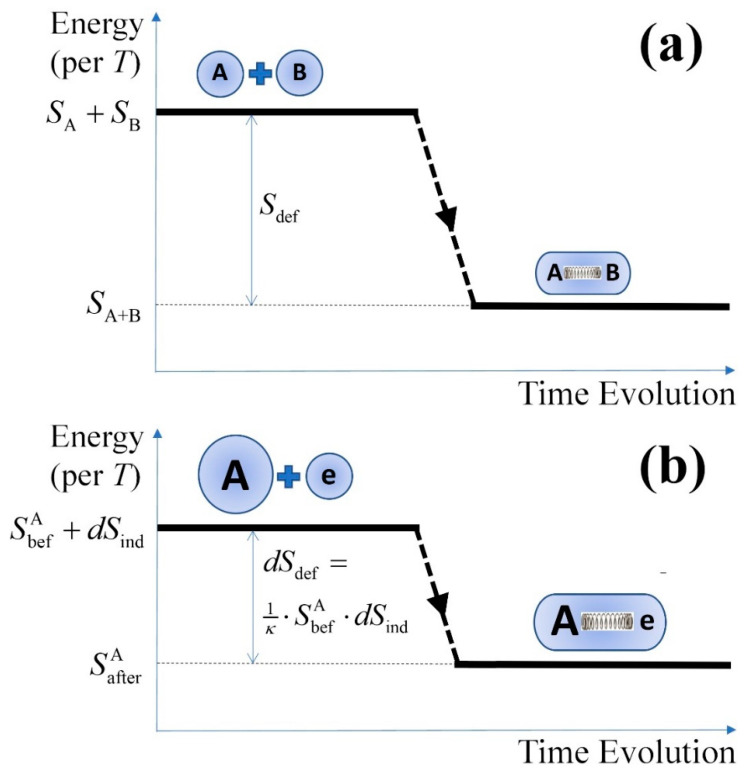
Diagram of entropy differences (equivalent to energy level per temperature) with respect to the time evolution during the composition of a system, when (**a**) it is composed by two subsystems A and B, as shown in Equations (5) and (6), and (**b**) an independent elementary subsystem is added to it, as shown in Equations (7) and (8). The entropy of the system with correlations is lower than the entropy of the system if there were no correlations, because the correlations add order to the system; at the composition of the system, its entropy differs from the sum of the subsystem entropies by the entropy defect.

Figure 2 illustrates the entropy defect in the total entropy of a system, when (a) it is composed by two subsystems A and B, as shown in Equations (5) and (6), and (b) an independent elementary subsystem is added to it, as shown in Equations (7) and (8).

Next, we solve Equations (10) and (11) to express the total nonextensive entropy *S* of a composed system, when this is constructed in the presence of correlations among its constituents. This entropy is expressed in terms of its extensive limit, *S*_ind_, that is, the entropy of the collection of all the elementary subsystems, which would have been the actual entropy *S* of the system if there were no correlations.

### 2.3. Connection with the Formalism of Kappa Distributions

#### 2.3.1. General

Here, we develop and solve the (difference/differential) equations of entropy that provide the total nonextensive entropy of the system *S*, expressed in terms of its extensive limit, *S*_ind_. We examine both the discrete and continuous descriptions and show that the formalism of kappa distributions appears naturally in the expression of the nonextensive entropy.

#### 2.3.2. Discrete Description

Equation (10) constitutes a 1D linear discrete map or difference equation (e.g., [6,44,45,46]), which can be solved to find the entropy *S_n_* for each *n*:

(13) $$S_{n}=(1-\frac{1}{\kappa }dS_{\mathrm{ind}})\cdot S_{n-1}+dS_{\mathrm{ind}},\;\mathrm{for}\;\mathrm{all}\;n=1,\;2,\;\ldots ,\;N.$$

It describes the entropy *S_n_* of a system, which is composed nonextensively of *n* elementary subsystems, each of entropy *dS*_ind_, when constructed by the interaction of a system with entropy *S_n_*_−1_ (composed nonextensively of *n*−1 elementary subsystems) and a single elementary subsystem. Equation (13) can be rewritten as:

(14) $$(1-\frac{1}{\kappa }S_{n})=(1-\frac{1}{\kappa }dS_{\mathrm{ind}})\cdot (1-\frac{1}{\kappa }S_{n-1}),\;\mathrm{for}\;\mathrm{all}\;n=1,\;2,\;\ldots ,\;N.$$

We set $x_{n}\equiv 1-\frac{1}{\kappa }S_{n}$ and $\alpha \equiv 1-\frac{1}{\kappa }dS_{\mathrm{ind}}$, and thus Equation (14) is written as $x_{n}=\alpha \cdot x_{n-1}=\alpha^{2}\cdot x_{n-2}$$=\alpha^{n}\cdot x_{0}$, where $x_{0}\equiv 1-\frac{1}{\kappa }S_{0}=1$, since there is no initial system and no entropy ($S_{0}=0$) existing before the very first mixing of elementary subsystems. Hence, $(1-\frac{1}{\kappa }S_{n})={\left(1-\frac{1}{\kappa }dS_{\mathrm{ind}}\right)}^{n}$, or given of Equation (9),

(15) $$(1-\frac{1}{\kappa }S_{n})={\left(1-\frac{1}{n\cdot \kappa }S_{\mathrm{ind}}{}_{n}\right)}^{n},\;\mathrm{for}\;\mathrm{all}\;n=0,\;1,\;\ldots ,\;N,$$

or

(16) $${\left(1-\frac{1}{\kappa }S_{n}\right)}^{-\kappa }={\left(1-\frac{1}{n\cdot \kappa }S_{\mathrm{ind}}{}_{n}\right)}^{-n\cdot \kappa },\;\mathrm{for}\;\mathrm{all}\;n=0,\;1,\;\ldots ,\;N.$$

Therefore, from Equation (15) we find that for a final number of *N* elementary subsystems involved in the final composed system, we have:

(17) $${\left(1-\frac{1}{\kappa }S_{N}\right)}^{-\kappa }={\left(1-\frac{1}{N\cdot \kappa }S_{\mathrm{ind}}{}_{N}\right)}^{-N\cdot \kappa },$$

and thus, the entropy of the system is given by:

(18) $$S_{N}=\kappa \cdot \left[1-{\left(1-\frac{1}{N\cdot \kappa }S_{\mathrm{ind}}{}_{N}\right)}^{N}\right].$$

We also recall the known notation of deformed exponential/logarithm functions:

(19) $$W\equiv \exp_{\kappa }(S_{N})=\exp_{\left(N\cdot \kappa \right)}(S_{\mathrm{ind}}{}_{N}),$$

which constitutes the multiplicity *W* (or statistical weight), that is, the number of microstates corresponding to a particular macrostate of a thermodynamic system. Then, we can solve in terms of entropies:

(20) $$S_{N}=\ln_{\kappa }\left[\exp_{\left(N\cdot \kappa \right)}(S_{\mathrm{ind}}{}_{N})\right]\;\Leftrightarrow \;S_{\mathrm{ind}}{}_{N}=\ln_{\left(N\cdot \kappa \right)}\left[\exp_{\kappa }(S_{N})\right],$$

where the *κ*- deformed exponential function and its inverse, the deformed logarithm function (e.g., see: [47,48,49,50]),

(21) $$\exp_{\kappa }(x)\equiv {\left(1-\frac{1}{\kappa }x\right)}^{-\kappa },\;\ln_{\kappa }(x)\equiv \kappa (1-x^{-\frac{1}{\kappa }}),$$

with

(22) $$\exp_{\kappa }[\ln_{\kappa }(x)]=\ln_{\kappa }[\exp_{\kappa }(x)]=x.$$

Here, we use the kappa index as a subscript for the notation of the deformed functions but note that the *q*-index could have been equivalently used (see also: Appendix A).

For a large number *N* of the involved elementary subsystems, *N* >> 1, and considering that the extensive entropy has a given fixed value, i.e., $dS_{\mathrm{ind}}{}_{N}=S_{\mathrm{ind}}{}_{N}/N$, we obtain:

(23) $${\left(1-\frac{1}{N\cdot \kappa }S_{\mathrm{ind}}{}_{N}\right)}^{-N\cdot \kappa }\to \exp(S_{\mathrm{ind}}{}_{N}).$$

**Figure 3:**
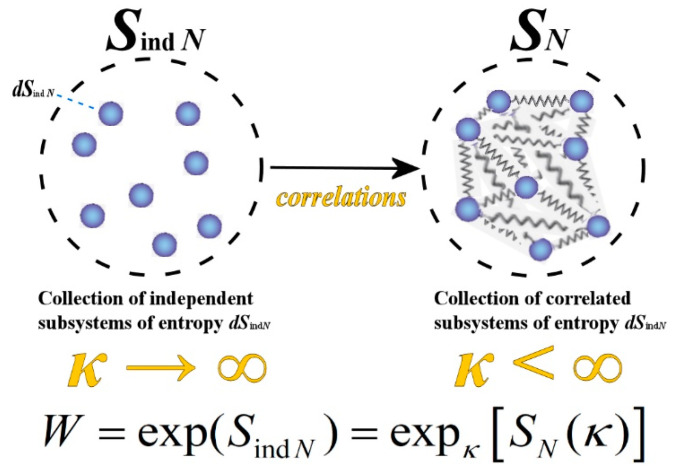
The relationship between the entropy of a system, *S_N_*, composed by *N* originally independent subsystems, and the respective extensive entropy, *S*_ind*N*_, that is, the sum of the subsystem entropies, *dS*_ind*N*_ = *S*_ind*N*_/*N*. The statistical weight W is independent of the kappa index, for a fixed value of the extensive entropy of the system *S*_ind*N*_ (i.e., for *dS*_ind*N*_∝1/*N*) and at the limit of *N* >> 1. The missing entropy, that is, the difference between the extensive *S*_ind*N*_ and the nonextensive *S_N_* entropic values, is caused by the presence of correlations and is interpreted and expressed as an entropy defect. As shown in Figure 2, the entropy of the system with correlations, *S_N_*, is lower than the entropy of the system if there were no correlations, *S*_ind*N*_, because the correlations add order to the system; the missing entropy gives the entropy defect.

Hence, Equations (17)–(20) can be, respectively, reduced, for a large *N*, as:

(24) $${\left(1-\frac{1}{\kappa }S_{N}\right)}^{-\kappa }=\exp(S_{\mathrm{ind}}{}_{N}),$$

(25) $$S_{N}=\kappa \cdot (1-e^{-\frac{1}{\kappa }\cdot S_{\mathrm{ind}}{}_{N}}),$$

(26) $$W=\exp_{\kappa }(S_{N})=\exp(S_{\mathrm{ind}}{}_{N}),$$

(27) $$S_{N}=\ln_{\kappa }\left[\exp(S_{\mathrm{ind}}{}_{N})\right]\;\Leftrightarrow \;S_{\mathrm{ind}}{}_{N}=\ln\left[\exp_{\kappa }(S_{N})\right],$$

where we observe that the multiplicity $W=\exp_{\kappa }(S_{N})$ becomes a thermodynamic quantity independent of the kappa index (see Figure 3). In addition, Equation (27) shows that a system, consisting of *N* elementary subsystems, has an entropy equal to *S*_ind *N*_ if the composition is constructed with no correlations (extensively) and entropy equal to *S_N_* if the composition is constructed with correlations (nonextensively).

#### 2.3.3. Continuous Description

Equation (11) can be solved directly, by integrating from Equation (11) the system’s entropy *dS* from 0 to *S* and elementary entropy *dS*_ind_ from 0 to *S*_ind_, obtaining:

(28) $${\left(1-\frac{1}{\kappa }S\right)}^{-\kappa }=\exp(S_{\mathrm{ind}}),$$

(29) $$S=\kappa \cdot (1-e^{-\frac{1}{\kappa }\cdot S_{\mathrm{ind}}}),$$

(30) $$W=\exp_{\kappa }(S)=\exp(S_{\mathrm{ind}}),$$

(31) $$S=\ln_{\kappa }\left[\exp(S_{\mathrm{ind}})\right]\;\Leftrightarrow \;S_{\mathrm{ind}}=\ln\left[\exp_{\kappa }(S)\right],$$

which are similar to Equations (24)–(27), respectively, when interpreting the entropy of a collection of *N* subsystems in a continuous description, namely, $S_{N}\to S=S(N)$ and $S_{\mathrm{ind}}{}_{N}\to S_{\mathrm{ind}}=S_{\mathrm{ind}}(N)$. Equations (18) (discrete description) and (25) or (29) (continuous description) completed the task of expressing the total nonextensive entropy of the system *S* in terms of its extensive limit, *S*_ind_.

It is also important that we produced the formalism of kappa distributions for the entropic form, starting only from thermodynamic definitions (compare with the entropic form for kappa distributions [3]). Section 4 will analyze further this result, expressing entropy in terms of both kappa and temperature.

## 3. Thermodynamic Definitions

### 3.1. Thermodynamic Definition of the Kappa Index

The entropy defect defines the kappa index within a thermodynamic context. Starting from Equation (8), which expresses the entropy defect of mixing a system of entropy *S* with an elementary independent subsystem of entropy *S*_ind_,

(32) $$dS_{\mathrm{def}}(S,dS_{\mathrm{ind}})=\frac{1}{\kappa }dS_{\mathrm{ind}}\cdot S,$$

we obtain the thermodynamic definition of the kappa:

(33) $$\frac{1}{\kappa }\equiv \frac{dS_{\mathrm{def}}}{SdS_{\mathrm{ind}}}.$$

We recall that we work in the framework of the canonical ensemble and fixed volume and number of particles. Hence, the involved derivatives are written as: $dS/dS_{\mathrm{ind}}\to {\left(\partial S/\partial S_{\mathrm{ind}}\right)}_{V,N}$ and $\partial S/\partial U\to {\left(\partial S/\partial U\right)}_{V,N}$. Hence, the definition becomes:

(34) $$\frac{1}{\kappa }\equiv \frac{1}{S}\cdot {\left(\frac{\partial S_{\mathrm{def}}}{\partial S_{\mathrm{ind}}}\right)}_{V,N}.$$

Thus, when adding an independent part of entropy *dS*_ind_ to a system with correlations among its constituents, its entropy *S* increases by a smaller amount, *dS* = *dS*_ind_ − *dS*_def_, where the difference is caused by the corresponding entropy defect *dS*_def_ that determines the thermodynamic value of kappa according to Equation (34).

The ratio of the entropy defect per entropy of the elementary independent subsystems that compose the total system, normalized to the entropy of the system, constitutes the thermodynamic definition of the inverse kappa (with the appropriate care of the defect sign); note the similarity of the thermodynamic definition of the inverse temperature.

The thermodynamic definition is actually used to derive the connection between nonextensive and extensive entropies (as shown in Equation (11) and found in Equation (29)), namely:

(35) $$\frac{1}{\kappa }=-\frac{\left(S+dS)-(S+dS_{\mathrm{ind}}\right)}{SdS_{\mathrm{ind}}}=\frac{1}{S}\cdot \left(1-\frac{dS}{dS_{\mathrm{ind}}}\right).$$

### 3.2. Thermodynamic Definition of the Temperature

Considering again the fixed volume and number of particles, Equation (11) becomes:

(36) $$1-\frac{1}{\kappa }S={\left(\frac{\partial S}{\partial S_{\mathrm{ind}}}\right)}_{V,N},$$

while the thermodynamic definition of temperature, Equation (3), is written as:

(37) $$\frac{1}{T}\equiv \frac{1}{1-\frac{1}{\kappa }S}\cdot {\left(\frac{\partial S}{\partial U}\right)}_{V,N}=\frac{{\left(\partial S/\partial U\right)}_{V,N}}{{\left(\partial S/\partial S_{\mathrm{ind}}\right)}_{V,N}}={\left(\frac{\partial S_{\mathrm{ind}}}{\partial U}\right)}_{V,N},$$

which is now expressed without involving of the independent thermodynamic property of the kappa index. Note that the expression connects the temperature with the extensive entropy of the system, corresponding to the collection of independent elementary subsystems. This matches the classical Clausius thermodynamic definition of temperature, where the entropy is extensive, $1/T\equiv {\left(\partial S/\partial U\right)}_{V,N}$.

We highlight the difference from the classical thermodynamic definition of temperature, which is the existence of the entropy defect, explicitly expressed in:

(38) $$\frac{1}{T}\equiv {\left(\frac{\partial S_{\mathrm{ind}}}{\partial U}\right)}_{V,N}={\left(\frac{\partial S}{\partial U}\right)}_{V,N}+{\left(\frac{\partial S_{\mathrm{def}}}{\partial U}\right)}_{V,N}.$$

### 3.3. Connection with the Kinetic Definitions

The kinetic definition of the temperature and the kappa index are, respectively, related to the particle kinetic energy expectation value [5] and correlations [43,51,52]. In particular, the temperature is kinetically defined by the mean particle kinetic energy 〈ε〉 per half degrees of freedom *D*, while the kappa index is kinetically defined by the correlation ρ of the kinetic energies among any two particles (see also: [23], Chapter 5, and [53,54]). Accompanying the kinetic and thermodynamic definitions, we have for the kappa:

(39) $$\frac{1}{\kappa }\equiv \frac{\rho }{\frac{1}{2}D},\;\frac{1}{\kappa }\equiv \frac{1}{S}\cdot {\left(\frac{\partial S_{\mathrm{def}}}{\partial S_{\mathrm{ind}}}\right)}_{V,N},$$

and the temperature:

(40) $$\frac{1}{T}\equiv \frac{\frac{1}{2}D}{\langle \varepsilon \rangle },\;\frac{1}{T}\equiv {\left(\frac{\partial S_{\mathrm{ind}}}{\partial U}\right)}_{V,N}.$$

An interesting combination of both definitions, canceling out the elementary entropy, is:

(41) $$\frac{1}{\kappa T}\equiv \frac{\rho }{\langle \varepsilon \rangle },\;\frac{1}{\kappa T}\equiv \frac{1}{S}\cdot {\left(\frac{\partial S_{\mathrm{def}}}{\partial U}\right)}_{V,N}.$$

Lastly, we can express directly the kinetic energy expectation value and correlation to the thermodynamic quantities:

(42) $$\rho \sim \frac{1}{S}\cdot {\left(\frac{\partial S_{\mathrm{def}}}{\partial S_{\mathrm{ind}}}\right)}_{V,N},\;\langle \varepsilon \rangle \sim {\left(\frac{\partial U}{\partial S_{\mathrm{ind}}}\right)}_{V,N},$$

with all entropies normalized to the half degrees of freedom (i.e., $S/(\frac{1}{2}D)\to S$). As expected, the particle kinetic energy expectation value and correlation are, respectively, proportional to the internal energy and entropy defect, per extensive entropy.

## 4. Entropic Formalism Associated with Kappa Distributions

In Section 2.3 and Equations (28)–(31), we derived the expression of the total nonextensive entropy of the system *S* in terms of its extensive limit, *S*_ind_. Since *S*_ind_ refers to an extensive collection of the elementary subsystems, it is independent of the kappa index, expressed as a function of the temperature. Indeed, we have:

(43) $$dS_{\mathrm{ind}}=dU/T=\frac{1}{2}D\cdot N\cdot dT/T=\frac{1}{2}D\cdot N\cdot d\ln(T/T_{0}),$$

or

(44) $$S_{\mathrm{ind}}=\frac{1}{2}D\cdot N\cdot \ln(T/T_{0}),$$

where the thermal constant *T*_0_ constitutes the minimum temperature for the entropy to be positive. In [55,56] and [23] (Chapters 2 and 5), this thermal constant was shown to be given by $k_{B}T_{0}=C\cdot \hbar_{c}^{2}{\left(m_{e}m_{i}\right)}^{-\frac{1}{2}}\cdot \lambda_{c}^{-2}\cdot g_{\kappa ,N}$, where *C* = (9π/2)^1/3^/*e* ≈ 0.89; λ_c_ is the smallest correlation length, which is interpreted by the interparticle distance *b*~*n*^−1/*d*^ for collisional particle systems (absence of correlations) or by the Debye length λ_D_ for collisionless particle systems (presence of local correlations) [57,58]. The factor *g_κ_*_,*N*_ depends on the kappa index κ and the number of correlated particles; for a large number of particles, it becomes *g_κ_*_,*N*_ ≈ 1. Finally, the phase-space parcel $\hbar_{c}^{}$ is typically given by the Planck’s constant, but it was shown to represent a different and larger constant in space plasmas, where Debye shielding limits the distance of correlations, $\hbar_{*}^{}=(1.19\pm 0.05)\times 10^{-22}J\cdot s$ [55,59,60,61,62,63] (namely, $\hbar_{c}^{}$ = ℏ, when no correlations exist among particles, and $\hbar_{c}^{}$ = $\hbar_{*}^{}$, when significant correlations exist among particles beyond their nearest neighbors, as in the case of the majority of space plasmas).

Combining now Equation (44), that is, the expression of the extensive entropy in terms of the temperature, *S*_ind_ (*T*), with Equation (18) or Equation (29), that is, the expression of the nonextensive *S* in terms of extensive *S*_ind_ entropies, we end up with the expression of the nonextensive entropy *S* in terms of the temperature, *S* (*T*).

For the discrete description, we obtain:

(45) $${\left(1-\frac{1}{\kappa }S_{N}\right)}^{-\kappa }={\left(1-\frac{1}{N\cdot \kappa }S_{\mathrm{ind}}{}_{N}\right)}^{-N\cdot \kappa }={\left[1-\frac{\frac{1}{2}D}{\kappa }\ln(T/T_{0})\right]}^{-N\cdot \kappa },$$

leading to

(46) $$S_{N}=\kappa \cdot \left\{1-{\left[1-\frac{1}{\kappa }\cdot \frac{1}{2}D\cdot \ln(T/T_{0})\right]}^{N}\right\}.$$

For the continuous description, we can write $d\ln{\left(T/T_{0}\right)}^{\frac{1}{2}D\cdot N}=dS_{\mathrm{ind}}=dS/(1-\frac{1}{\kappa }S)$$=d[\ln{\left(1-\frac{1}{\kappa }S\right)}^{-\kappa }]$ and obtain:

(47) $${\left(1-\frac{1}{\kappa }S\right)}^{-\kappa }\equiv \exp_{\kappa }(S)=\exp(S_{\mathrm{ind}})={\left(T/T_{0}\right)}^{\frac{1}{2}D\cdot N},$$

leading to

(48) $$S=\kappa \cdot \left(1-e^{-\frac{1}{\kappa }\cdot S_{\mathrm{ind}}}\right)=\kappa \cdot \left[1-{\left(T/T_{0}\right)}^{-\frac{1}{\kappa }\cdot \frac{1}{2}D\cdot N}\right],$$

or

(49) $$S\equiv \ln_{\kappa }\left[\exp(S_{\mathrm{ind}})\right]=\ln_{\kappa }\left[{\left(T/T_{0}\right)}^{\frac{1}{2}D\cdot N}\right].$$

Note that we may arrive at the continuous description and Equations (47) and (48), starting from the discrete description and Equations (45) and (46). Indeed, for *N* >> 1, and considering that the extensive entropy has a given fixed value, i.e., $S_{\mathrm{ind}}{}_{N}\neq S_{\mathrm{ind}}{}_{N}(N)$ and $dS_{\mathrm{ind}}{}_{N}=S_{\mathrm{ind}}{}_{N}/N$, we have ${\left\{1-\frac{1}{N}\cdot \left[\frac{1}{\kappa }\cdot \frac{1}{2}D\cdot N\cdot \ln(T/T_{0})\right]\right\}}^{N}$→$\exp\left[-\frac{1}{\kappa }\cdot \frac{1}{2}D\cdot N\cdot \ln(T/T_{0})\right]$$={\left(T/T_{0}\right)}^{-\frac{1}{\kappa }\cdot \frac{1}{2}D\cdot N}$.

We have ended up with the nonextensive entropy as a function of the independent thermodynamic properties of the kappa index and temperature. This temperature–entropy relationship is associated with kappa distributions and generalizes the Sackur–Tetrode entropic formula [64,65]. This is derived as follows. First, we substitute from Equations (46) and (48) the thermal constant *T*_0_, defined in Equation (44) as $k_{B}T_{0}=C\cdot \hbar_{c}^{2}{\left(m_{e}m_{i}\right)}^{-\frac{1}{2}}\cdot \lambda_{c}^{-2}\cdot g_{\kappa ,N}$, with $g_{\kappa ,N}≅1$, $\lambda_{c}^{}=b=n^{-\frac{1}{3}}$, $\hbar_{c}^{}=\hbar$, and we find, respectively:

(50) $$S_{N}(n,T,N,\kappa )=\kappa \cdot \left\{1-{\left\{1-\frac{1}{\kappa }\cdot \ln\left\{{\left[C\cdot k_{B}^{-1}\hbar^{2}{\left(m_{e}m_{i}\right)}^{-\frac{1}{2}}\right]}^{-\frac{3}{2}}\cdot \frac{T^{\frac{3}{2}}}{n}\right\}\right\}}^{N}\right\},$$

and

(51) $$S(n,T,N,\kappa )=\kappa \cdot \left\{1-{\left\{{\left[C\cdot k_{B}^{-1}\hbar^{2}{\left(m_{e}m_{i}\right)}^{-\frac{1}{2}}\right]}^{-\frac{3}{2}}\cdot \frac{T^{\frac{3}{2}}}{n}\right\}}^{-\frac{1}{\kappa }\cdot N}\right\}.$$

Then, we express Equation (50) or Equation (51) in terms of the number of particles *N*, internal energy $U=\frac{3}{2}N\cdot k_{B}T$, and volume V=N/n, following the steps shown in the direct derivation from kappa distributions in [3] (see also: [66]):

(52) $$S_{N}(V,T,N,\kappa )=\kappa \cdot \left\{1-{\left\{1-\frac{1}{\kappa }\cdot \ln\left\{{\left[\frac{3}{2}C\cdot \hbar^{2}{\left(m_{e}m_{i}\right)}^{-\frac{1}{2}}\right]}^{-\frac{3}{2}}\cdot \frac{U^{\frac{3}{2}}V}{N^{\frac{5}{2}}}\right\}\right\}}^{N}\right\},$$

and

(53) $$S(V,T,N,\kappa )=\kappa \cdot \left\{1-{\left\{{\left[\frac{3}{2}C\cdot \hbar^{2}{\left(m_{e}m_{i}\right)}^{-\frac{1}{2}}\right]}^{-\frac{3}{2}}\cdot \frac{U^{\frac{3}{2}}V}{N^{\frac{5}{2}}}\right\}}^{-\frac{1}{\kappa }\cdot N}\right\}.$$

Lastly, we comment on the requirement of global extensivity at the thermodynamic limit (*N*→∞) that may characterize a macroscopic system and the difference between global extensivity and local nonextensivity. Systems with correlations among its constituents are characterized by a nonextensive entropy, as shown in Equation (5), with the involved defect described by Equation (6). Nevertheless, the length scale of correlations is typically finite. For instance, the Debye length in plasmas characterizes the scale over which the electrons and ions are electrostatically self-shielding. Systems with sizes significantly larger than their correlation length can be understood as an ensemble of “correlation clusters”, where each cluster includes the constituents within the size of the correlation length scale. While correlations exist between the constituents of each cluster, the constituents of different clusters are independent. Therefore, the entropy of each cluster is nonextensive and thus not proportional to the entropy of each constituent. However, the entropy of the system is proportional to the number of correlation clusters and to the nonextensive entropy of each cluster. Thus, the system is globally extensive due to the independence of correlation clusters. (See also: [55,59].)

## 5. Total Entropy Defect Associated with the System’s Composition

Here, we formulate the nonextensive entropy of a system composed by a number *N* of subsystems with correlations and express it in terms of the respective extensive entropy and temperature.

Let the system be composed by *N* subsystems with correlations, with entropy $S_{N}=S_{N}(\kappa )$. If there were no correlations in the system, its entropy would have been *S*_ind*N*_, that is, the entropy of an extensive collection of *N* subsystems, namely, $S_{\mathrm{ind}}{}_{N}=S_{N}(\kappa \to \infty )$, or simply noted by $S_{N}(\infty )$. The relationship between the two entropies, as given by Equations (17) or (18), is:

(54) $$\exp_{\kappa }\left[S_{N}(\kappa )\right]=\exp_{\left(N\cdot \kappa \right)}\left[S_{N}(\infty )\right],$$

or

(55) $${\left[1-\frac{1}{\kappa }\cdot S_{N}(\kappa )\right]}^{-\kappa }={\left[1-\frac{1}{N\cdot \kappa }S_{N}(\infty )\right]}^{-N\cdot \kappa },$$

which can lead to the expression of *S_N_* (*κ*):

(56) $$S_{N}(\kappa )=\kappa \cdot \left\{1-{\left[1-\frac{1}{N\cdot \kappa }\cdot S_{N}(\infty )\right]}^{N}\right\}.$$

The total entropic defect of the composed system, which stands for the total entropy reduction due to the order generated by the presence of all the involved correlations, is simply given by the entropy difference:

(57) $$S_{N}(\kappa )-S_{N}(\infty )=-S_{\mathrm{def},T}.$$

Its (algebraic) value can be expressed and normalized to the system’s entropy:

(58) $$f\equiv \frac{S_{\mathrm{def},T}}{S_{N}(\infty )}=1+\frac{\kappa }{S_{N}(\infty )}\cdot \left\{{\left[1-\frac{1}{N}\cdot \frac{S_{N}(\infty )}{\kappa }\right]}^{N}-1\right\}=f\left(\frac{\kappa }{S_{N}(\infty )};N\right),$$

where we define the function:

(59) $$f(x;N)\equiv 1+x\cdot \left\{{\left(1-\frac{1}{N\cdot x}\right)}^{N}-1\right\}=1+x\cdot \left\{\exp_{N}^{-1}(-\frac{1}{x})-1\right\}.$$

Note, if the kappa index is smaller than the extensive entropy per particle, $\kappa \leq \frac{1}{N}S_{N}(\infty )]$, then, the base involved in the formulation is negative, and the Tsallis cut-off condition [20,31] is in effect. According to this, the base-exponent quantity is set to zero:

(60) $$f(x;N)=1-x,\;\mathrm{for}\;x\equiv \frac{\kappa }{S_{N}(\infty )}\leq \frac{1}{N}.$$

The stronger the interactions among the subsystems and the induced correlations, the larger the entropy defect. The larger value of the function *f* in Equation (59) corresponds to values of $\kappa /S_{N}(\infty )$ with a more strongly bonded system by means of stronger correlations; all expressions are functions of the number of composing subsystems *N*.

**Figure 4:**
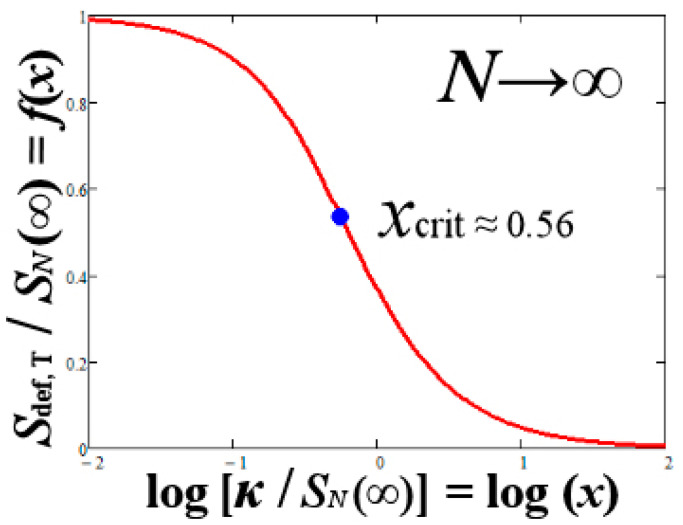
Plot of the function *f* with respect to the logarithm of $x\equiv \kappa /S_{N}(\infty )$, as shown in Equation (59). This function gives the total entropic defect of a composed system, normalized to the total entropy, for a large number *N*. (The critical value corresponds to the point of inflection, which is where the defect decreases at a slower rate with increasing of log (*x*)).

Figure 4 plots this function with respect to *x* = $\kappa /S_{N}(\infty )$. The critical point where the defect decreases at a slower rate with the increase of the kappa index is given by the point of inflection with respect to log (*x*); this is given by *x*_crit_ = [$\kappa /S_{N}(\infty )$]_crit_ ≅ 0.56.

Substituting from Equation (44) and taking into account also the kappa–correlation formula in Equation (39), we obtain the following optimal kappa index–temperature relationship:

(61) $$\kappa \sim 0.56\cdot \frac{1}{2}D\cdot \ln(T/T_{0}),$$

corresponding to the correlation–temperature formula:

(62) $$\rho \sim 1.8\cdot {\left[\ln(T/T_{0})\right]}^{-1}.$$

This relationship justifies the expectation that temperature, which is a measure of thermal random fluctuations, destroys correlations among particles [57,58].

It is interesting to compare this *κ*–*T* relationship with those observed in space plasmas (see: [22,23,67,68,69,70,71,72,73]). Such a relationship was remotely observed between the measurements of the kappa index and the temperature of the largely proton plasma in the inner heliosheath, between the termination shock and heliopause. Using energetic neutral atom (ENA) observations from the Interstellar Boundary Explorer (IBEX) [74,75] with energies above ∼0.7 keV [76], and by connecting the observed ENA flux to the kappa distribution of velocities of the source protons, [68] produced the sky maps of the (radially) average values of temperature and kappa, as well as of other thermodynamic quantities of the ENA-source proton plasma in the inner heliosheath (see also: [69,77,78]). Positive correlations characterize the measurements of the kappa index and the logarithm of temperature.

Moreover, we note that a *κ*–*T* relationship does not break the independence between temperature and the kappa index. First, the bi-parametric dependence of entropy *S* (*κ*,*T*), or of the entropy defect *f*, may be reversed to have the kappa expressed in terms of the other two arguments, i.e., *κ* (*S*,*T*) or *κ* (*f*,*T*); then, *S* or *f* is kept fixed (e.g., fixed at their maximum or inflection point).

## 6. Conclusions

In this study, we developed the explicit thermodynamic definitions of the temperature and kappa index and showed their connection with the formalism of nonextensive statistical mechanics and kappa distributions. This allowed us to define the entropy defect in the composition of a system, which leads to a nonextensive characterization of the entropy of the composed system that differs from the entropy of the system in its extensive limit. In particular, a system is composed extensively when its elementary subsystems interact with no correlations, as if they were independent, leading to no entropy defect and to an extensive entropy (the entropy of the whole system sums the entropies of the elementary subsystems). However, a system is composed nonextensively when its elementary subsystems are connected through interactions that can induce correlations (e.g., long-range interactions), leading to an entropy defect and a nonextensive property of the entropy.

The missing entropy, that is, the difference between the extensive and the nonextensive entropic values, is caused by the presence of correlations and is interpreted and expressed as an entropy defect. Thus, the entropy of the system with correlations is lower than the entropy of the system if there were no correlations, because the correlations add order to the system, and the missing entropy gives the entropy defect.

In summary, the paper develops the following theoretical aspects:

(i) The thermodynamic definition of the temperature is now consistent and well-stated, since it involves no other independent thermodynamic parameter, such as the kappa index.
(ii) The introduction of the entropy defect, which quantifies the amount of entropy associated with correlations in the assembly of a system, similarly to how a mass defect quantifies the energy associated with the assembly of subatomic particles.
(iii) The relationship between the entropy defect and the kappa is now clearly stated for any individual or composed system and independent of the temperature, leading to a consistent thermodynamic definition of the kappa index.
(iv) The developed thermodynamic definitions of kappa and temperature are connected with the formalism of kappa distributions, as well as the corresponding kinetic definitions of temperature and kappa index that originated from the kappa distributions.
(v) The entropy of a system characterized by correlations is determined using both discrete and continuous descriptions; the resulted entropic form generalizes the Sackur–Tetrode entropic formula.
(vi) The nonextensive entropy of a system composed by a number of subsystems with correlations is expressed in terms of the kappa index, the temperature, and the number of the involved subsystems; the same holds for the entropy defect, that is, the difference between the extensive and nonextensive entropies.
(vii) The total entropy defect of the system is used to find the relationship among the involved thermodynamic parameters, an optimal relationship between kappa and temperature is deduced, and the correlation coefficient is shown to be inversely proportional to the temperature logarithm.

Having brought the analogue of the classical mass defect to the concept of entropy and thermodynamics, it is then straightforward to apply the developed formalism and theoretical findings on the entropic defect to systems of charged particles in magnetic fields such as in space plasmas, where Debye shielding and magnetic fields can induce correlations among particles [33]. It is interesting that there may be examples that could be experimentally testable, which could include colloids and dusty plasmas with confinement [79,80,81].

Finally, in Table 1 we provide the basic equations and formulae derived in this paper.

## Figures and Tables

**Table 1:** Table of the basic derived equations and formulae and their explanations.

Equations and Formulae	Explanation
$S_{A+B}=S_{A}+S_{B}-S_{\mathrm{def}}(S_{A},S_{B})$	Non-additive partitioning rule
$S_{\mathrm{def}}(S_{A},S_{B})=\frac{1}{\kappa }\cdot S_{A}\cdot S_{B}$	Entropy defect; this is proportional to the combining entropies $S_{A}$ and $S_{B}$; the proportionality constant defines kappa
$S_{\mathrm{ind}}{}_{N}$ or $S_{N}(\kappa \to \infty )$	Entropy of a system with no correlations among its constituents
$S_{N}$ or $S_{N}(\kappa )$	Entropy of a system with correlations among its constituents
$\exp_{\kappa }(S_{N})=\exp_{\left(N\cdot \kappa \right)}(S_{\mathrm{ind}}{}_{N})$ or $\begin{array}{c} S_{N}(\kappa )=\kappa \cdot \left\{1-\exp_{N}\left[-\frac{1}{\kappa }S_{N}(\infty )\right]\right\}\\ =\kappa \cdot \left\{1-{\left[1-\frac{1}{N\kappa }\cdot S_{N}(\infty )\right]}^{N}\right\} \end{array}$	Connection between the nonextensive entropy (actual entropy of a system with correlations) and the extensive entropy (entropy of the system if there were no correlations)
$S_{N}=\ln_{\kappa }[\exp(S_{\mathrm{ind}}{}_{N})]\Leftrightarrow S_{\mathrm{ind}}{}_{N}=\ln[\exp_{\kappa }(S_{N})]$ or $S_{N}(\kappa )=\kappa \cdot \left\{1-\exp\left[-\frac{1}{\kappa }S_{N}(\infty )\right]\right\}$	Connection between the entropies for a large number of constituents, *N* >> 1
$S_{\mathrm{def},T}\equiv S_{N}(\infty )-S_{N}(\kappa )$	Total entropy defect combined from all the constituents
$\frac{S_{\mathrm{def},T}}{S_{N}(\infty )}=f(\frac{\kappa }{S_{N}(\infty )})$, $f(x)\equiv 1+x\cdot [{\left(1-\frac{1}{N\cdot x}\right)}^{N}-1]$	Total entropy defect and its dependence on kappa
$S_{\mathrm{ind}}{}_{N}=\frac{1}{2}D\cdot N\cdot \ln(T/T_{0})$	Extensive entropy in terms of temperature
$S_{N}=\kappa \cdot \left\{1-{\left[1-\frac{1}{\kappa }\cdot \frac{1}{2}D\cdot \ln(T/T_{0})\right]}^{N}\right\}$ or $S_{N}=\kappa \cdot \left[1-{\left(T/T_{0}\right)}^{-\frac{1}{\kappa }\cdot \frac{1}{2}D\cdot N}\right]$, for *N* >> 1	Nonextensive entropy in terms of temperature
$k_{B}T_{0}=C\cdot \hbar_{c}^{2}{\left(m_{e}m_{i}\right)}^{-\frac{1}{2}}\cdot \lambda_{c}^{-2}\cdot g_{\kappa ,N}$, where *C* = (9π/2)^1/3^/e ≈ 0.89, and $g_{\kappa ,N}={\left[\frac{\Gamma (\kappa_{0})}{\sqrt{\frac{2\pi }{\kappa_{0}}}{\left(\frac{\kappa_{0}}{e}\right)}^{\kappa_{0}}}\cdot \frac{\sqrt{\frac{2\pi }{\kappa }}{\left(\frac{\kappa }{e}\right)}^{\kappa }}{\Gamma (\kappa )}\cdot \sqrt{\frac{\kappa }{\kappa_{0}}}\right]}^{-\frac{2}{3}\cdot \frac{1}{N}}\sim 1$, with $\kappa_{0}=\kappa -\frac{1}{2}D\cdot N$	Minimum temperature, *T* > *T*_0_, for the entropy to be positive; *k*_B_: Boltzmann constant, *m*_i_ and *m*_e_: ion and electron masses; λ_c_: smallest correlation length, that is, the interparticle distance for collisional particle systems (absence of correlations), or by the Debye length for collisionless particle systems (presence of local correlations); the phase-space parcel $\hbar_{c}^{}$ is given by the Planck’s constant, but it represents a larger constant in space plasmas.
$\frac{1}{T}\equiv {\left(\frac{\partial S_{\mathrm{ind}}}{\partial U}\right)}_{V,N}$ and $\frac{1}{T}\equiv \frac{\frac{1}{2}D}{\langle \varepsilon \rangle }$	Thermodynamic and kinetic definitions of the (inverse) temperature
$\frac{1}{\kappa }\equiv \frac{1}{S}\cdot {\left(\frac{\partial S_{\mathrm{def}}}{\partial S_{\mathrm{ind}}}\right)}_{V,N}$ and $\frac{1}{\kappa }\equiv \frac{\rho }{\frac{1}{2}D}$	Thermodynamic and kinetic definitions of the (inverse) kappa
$\frac{1}{\kappa T}\equiv \frac{\rho }{\langle \varepsilon \rangle }$, $\frac{1}{\kappa T}\equiv \frac{1}{S}\cdot {\left(\frac{\partial S_{\mathrm{def}}}{\partial U}\right)}_{V,N}$	Thermodynamic and kinetic definitions of the (inverse) product of kappa and temperature
$\langle \varepsilon \rangle \sim {\left(\frac{\partial U}{\partial S_{\mathrm{ind}}}\right)}_{V,N}$, $\rho \sim \frac{1}{S}\cdot {\left(\frac{\partial S_{\mathrm{def}}}{\partial S_{\mathrm{ind}}}\right)}_{V,N}$	Kinetic definitions expressed in terms of thermodynamic definitions
$\kappa \sim 0.56\cdot \frac{1}{2}D\cdot \ln(T/T_{0})$ or $\rho \sim 1.8\cdot {\left[\ln(T/T_{0})\right]}^{-1}$	The entropy maximization leads to a linear relationship between the kappa and temperature logarithm; the correlation coefficient is inversely proportional to the temperature (per *T*_0_) logarithm
$\exp_{\kappa }(x)\equiv {\left(1-\frac{1}{\kappa }x\right)}^{-\kappa }$, $\ln_{\kappa }(x)\equiv \kappa (1-x^{-\frac{1}{\kappa }})$ with $\exp_{\kappa }[\ln_{\kappa }(x)]=\ln_{\kappa }[\exp_{\kappa }(x)]=x$	Definitions of the *q*-deformed exponential and logaritm (inverse) functions

## Appendix A. Expressions in Terms of the Entropic *q*-Index

Nonextensive statistical mechanics are based on a generalized entropic formula [18] and the concept of the escort probability distributions [82], both characterized by the entropic parameter *q*-index. On the other hand, the formalism of kappa distributions and their statistical framework is characterized by the kappa index [23]. The equivalence between these two indices has already been established [5]:

(A1) κ=1/(q−1)⇔q=1+1/κ.

We stress that the differences between kappa distributions and the nonextensive distributions are simply a matter of terminology: the relationship between the kappa and *q*-index parameters is a simple substitution of variables. The kappa distribution and the *q*-Gaussian distribution are essentially the same, the concepts of the loss or gain of entropy after mixing are the same for the formalism described either in terms of kappa or *q*-index.

In Table A1, we provide all the basic equations and formulae derived in this paper, expressed in terms of the *q*-index (parallel to Table 1 expressed in terms of the kappa.)

**Table A1:** Table of the basic derived equations and formulae, expressed in terms of the *q*-index, and their explanations.

Equations and Formulae	Explanation
$S_{A+B}=S_{A}+S_{B}-S_{\mathrm{def}}(S_{A},S_{B})$	Non-additive partitioning rule
$S_{\mathrm{def}}(S_{A},S_{B})=(q-1)\cdot S_{A}\cdot S_{B}$	Entropy defect; this is proportional to the combining entropies $S_{A}$ and $S_{B}$
$S_{\mathrm{ind}}{}_{N}$ or $S_{N}(q\to 1)$	Entropy of a system with no correlations among its constituents
$S_{N}$ or $S_{N}(q)$	Entropy of a system with correlations among its constituents
$\exp_{q}(S_{N})=\exp_{1+(q-1)/N}(S_{\mathrm{ind}}{}_{N})$ or $\begin{array}{c} S_{N}(q)=\left\{1-\exp_{1+1/N}\left[-(q-1)S_{N}(\infty )\right]\right\}/(q-1)\\ =\left\{1-{\left[1-\frac{q-1}{N}\cdot S_{N}(\infty )\right]}^{N}\right\}/(q-1) \end{array}$	Connection between the nonextensive entropy (actual entropy of a system with correlations) and the extensive entropy (entropy of the system if there were no correlations)
$S_{N}=\ln_{q}[\exp(S_{\mathrm{ind}}{}_{N})]\Leftrightarrow S_{\mathrm{ind}}{}_{N}=\ln[\exp_{q}(S_{N})]$ or $S_{N}(q)=\left\{1-\exp\left[-(q-1)\cdot S_{N}(\infty )\right]\right\}$	Connection between the entropies for a large number of constituents, *N* >> 1
$S_{\mathrm{def},T}\equiv S_{N}(1)-S_{N}(q)$	Total entropy defect combined from all the constituents
$\frac{S_{\mathrm{def},T}}{S_{N}(1)}=f[\frac{1}{S_{N}(1)\cdot (q-1)}]$, $f(x)\equiv 1+x\cdot [{\left(1-\frac{1}{N\cdot x}\right)}^{N}-1]$	Total entropy defect dependence on the *q*-index
$S_{\mathrm{ind}}{}_{N}=\frac{1}{2}D\cdot N\cdot \ln(T/T_{0})$	Extensive entropy in terms of temperature
$S_{N}=\left\{1-{\left[1-(q-1)\cdot \frac{1}{2}D\cdot \ln(T/T_{0})\right]}^{N}\right\}/(q-1)$ or $S_{N}=\left[1-{\left(T/T_{0}\right)}^{-(q-1)\cdot \frac{1}{2}D\cdot N}\right]/(q-1)$, for *N* >> 1	Nonextensive entropy in terms of temperature
$k_{B}T_{0}=C\cdot \hbar_{c}^{2}{\left(m_{e}m_{i}\right)}^{-\frac{1}{2}}\cdot \lambda_{c}^{-2}\cdot g_{q,N}$, with *C* = (9π/2)^1/3^/e ≈ 0.89, and $g_{q,N}\sim 1$	Minimum temperature for the entropy to be positive
$\frac{1}{T}\equiv {\left(\frac{\partial S_{\mathrm{ind}}}{\partial U}\right)}_{V,N}$ and $\frac{1}{T}\equiv \frac{\frac{1}{2}D}{\langle \varepsilon \rangle }$	Thermodynamic and kinetic definitions of the (inverse) temperature
$q\equiv 1+\frac{1}{S}\cdot {\left(\frac{\partial S_{\mathrm{def}}}{\partial S_{\mathrm{ind}}}\right)}_{V,N}$ and $q\equiv 1+\frac{\rho }{\frac{1}{2}D}$	Thermodynamic and kinetic definitions of the *q*-index
$\frac{q-1}{T}\equiv \frac{\rho }{\langle \varepsilon \rangle }$, $\frac{q-1}{T}\equiv \frac{1}{S}\cdot {\left(\frac{\partial S_{\mathrm{def}}}{\partial U}\right)}_{V,N}$	Thermodynamic and kinetic definitions of the product of the index (*q*-1) and (inverse) temperature
$\langle \varepsilon \rangle \sim {\left(\frac{\partial U}{\partial S_{\mathrm{ind}}}\right)}_{V,N}$, $\rho \sim \frac{1}{S}\cdot {\left(\frac{\partial S_{\mathrm{def}}}{\partial S_{\mathrm{ind}}}\right)}_{V,N}$	Kinetic definitions expressed in terms of thermodynamic definitions
${\left(q-1\right)}^{-1}\sim 0.56\cdot \frac{1}{2}D\cdot \ln(T/T_{0})$ or $\rho \sim 1.8\cdot {\left[\ln(T/T_{0})\right]}^{-1}$	The entropy maximization leads to a linear relationship between the *q*-index and temperature logarithm; the correlation coefficient is inversely proportional to the temperature (per *T*_0_) logarithm
$\exp_{q}(x)\equiv {\left[1-(q-1)x\right]}^{\frac{1}{1-q}}$, $\ln_{q}(x)\equiv (1-x^{1-q})/(q-1)$ with $\exp_{q}[\ln_{q}(x)]=\ln_{q}[\exp_{q}(x)]=x$	Definitions of the *q*-deformed exponential and logaritm (inverse) functions
